# Perceived physical literacy and academic procrastination among college students: a chain mediation model of self-control resource depletion and psychological resilience

**DOI:** 10.3389/fpsyg.2026.1748126

**Published:** 2026-03-27

**Authors:** Weijie Zhang, Yanyun Gu, Jiahui Yu, Guohao Liu, Jingjing Li, Bing Liu

**Affiliations:** 1 Physical Education College, Shanghai University, Shanghai, China; 2 Shanghai Cao Yang No. 2 High School, Shanghai, China; 3 Physical Education Research Center, Shanghai University, Shanghai, China

**Keywords:** academic procrastination, college students, perceived physical literacy, psychological resilience, self-control resource depletion

## Abstract

**Background:**

Academic procrastination has become increasingly common among Chinese university students and now represents a practical challenge to the quality of higher education talent cultivation. As an important foundation for fostering positive psychological qualities and behavioral regulation among college students, physical literacy has received increasing attention for its potential role in alleviating academic procrastination.

**Methods:**

This study employed the Perceived Physical Literacy Scale, the College Students’ Academic Procrastination Scale, the Brief Self-Control Depletion Scale, and the Connor–Davidson Resilience Scale to investigate 951 students from two comprehensive universities in Shanghai, examining the effect of perceived physical literacy on academic procrastination and its underlying mechanisms.

**Results:**

(1) perceived physical literacy was significantly negatively correlated with academic procrastination and self-control resource depletion, whereas it was significantly positively correlated with psychological resilience, with correlation coefficients of −0.176, −0.121, and 0.188, respectively (all *p* < 0.01); (2) perceived physical literacy significantly negatively predicted academic procrastination among university students (*β* = −0.127, *p* < 0.01); (3) self-control resource depletion and psychological resilience each played a partial mediating role in the relationship between perceived physical literacy and academic procrastination, accounting for 20.39% and 15.53% of the total effect, respectively; and (4) self-control resource depletion and psychological resilience also exerted a significant chain mediating effect in the relationship between perceived physical literacy and academic procrastination.

**Conclusion:**

These findings indicate that perceived physical literacy can inhibit academic procrastination among university students by reducing self-control resource depletion and enhancing psychological resilience. This study provides both a theoretical basis and practical implications for universities seeking to strengthen physical practice, enhance students’ perceived physical literacy, and promote normative academic behavior.

## 1 Introduction

Academic procrastination has become an increasingly prevalent learning-related problem among Chinese university students and is gradually emerging as a salient practical issue that affects both the quality of talent cultivation and the effectiveness of university governance. As a maladaptive pattern of academic behavior, academic procrastination not only disrupts the normal instructional order and training process, but may also exert lasting adverse effects on students’ academic adjustment, career preparation, social integration, and mental health (González-Brignardello et al., 2023; Hamdan-Mansour et al., 2024). Existing research has consistently shown that procrastination is a form of self-regulatory failure and is associated with poorer academic performance, diminished well-being, and elevated risks of depression, anxiety, and stress (Boncales, 2019; Kim and Seo, 2015; Steel, 2007). In the context of contemporary Chinese higher education, this problem may be further exacerbated by intensifying competition in employment and academic evaluation (Liu et al., 2024; Zheng and Yan, 2024), growing demands for autonomous self-management after entering university (Wang et al., 2021), and the rapid penetration of internet-based media environments that amplify distraction and preferences for immediate gratification (Qi et al., 2025). Accordingly, identifying the antecedents of academic procrastination and clarifying possible intervention pathways has become an important task in research on student mental health promotion and academic governance.

Against this background, sport and physical activity have increasingly been viewed as important pathways for promoting university students’ social adaptation, psychological development, and behavioral optimization. Compared with a narrow focus on exercise frequency or intensity, perceived physical literacy may better capture individuals’ integrated psychological perceptions and competence-related experiences developed through participation in physical activity, including their self-evaluations of movement competence, the value they attach to sport, behavioral persistence, and lifelong engagement tendencies (Edwards et al., 2017; Sum et al., 2018; Sum et al., 2016; Whitehead, 2010). As a multidimensional quality characterized by embodied practice, rule-guided participation, situational interaction, and feedback-based reinforcement, perceived physical literacy may shape not only whether university students are willing to sustain engagement in physical activity, but also deeper psychological processes such as self-management, emotional regulation, and goal persistence (Ma et al., 2021). Therefore, examining the mechanisms underlying academic procrastination from the perspective of perceived physical literacy may extend the explanatory scope of procrastination research and provide a new practical basis for universities seeking to foster students’ psychological well-being and academic development through sport.

Self-regulation theory provides an important theoretical lens for understanding the relationship between perceived physical literacy and academic procrastination. This perspective suggests that individuals with stronger self-regulatory capacities are better able to maintain attention to long-term goals, manage emotions, and control behavior, thereby constraining and optimizing their current actions. From this perspective, academic procrastination can be understood as a typical manifestation of self-regulatory dysfunction in learning contexts (Steel, 2007). University students with higher levels of perceived physical literacy are more likely to possess clearer goal awareness, more stable behavioral persistence, and more positive self-evaluations, because physical literacy is conceptualized as a multidimensional capacity integrating motivation, confidence, competence, and knowledge/understanding that supports sustained engagement over the life course (Edwards et al., 2017; Whitehead, 2010). In measurement work, perceived physical literacy has been operationalized as a subjective, multidimensional construct capturing perceived competence, knowledge/understanding, and value-related perceptions that reflect individuals’ confidence and persistence in physical activity contexts (Sum et al., 2018; Sum et al., 2016). The rule awareness, task engagement, and delayed-gratification experiences accumulated through physical activity may generalize to non-sport domains, consistent with the broader literature on life-skill and self-regulation development through sport (Danish et al., 2011; Gould and Carson, 2008), thereby strengthening self-control and reducing tendencies toward task avoidance; related evidence in Chinese university students also shows that physical activity is associated with lower academic procrastination via self-control (Li et al., 2022). Meanwhile, embodied cognition theory further suggests that cognition is deeply rooted in bodily experience and shaped by the coordinated involvement of perception, action, and situational interaction (Wilson, 2002). As a typical form of embodied practice, physical activity not only enhances bodily activation and individuals’ experiences of movement, rhythm, control, and environmental feedback, but may also gradually reshape cognitive processing patterns and psychological regulation capacities through sustained bodily participation (Barsalou, 2008; Wilson, 2002). Thus, perceived physical literacy should not be understood merely as a sport-related cognitive evaluation; rather, it may influence psychological qualities and learning behaviors through the accumulation of embodied experiences (Edwards et al., 2017; Whitehead, 2010).

Nevertheless, the influence of perceived physical literacy on academic procrastination is unlikely to be a simple direct process. Prior research suggests that procrastination is closely related to deficits in self-control and adaptive psychological functioning, while resilience serves as an important protective factor against maladaptive academic behaviors (Huang et al., 2023; Zhang et al., 2024). According to the limited-resource model of self-control, self-regulation depends on finite psychological resources; when these resources are chronically depleted and insufficiently replenished, individuals become more prone to impulsive decision-making, task avoidance, and delayed action (Muraven et al., 1998). In this sense, academic procrastination is closely tied to insufficient self-control resources. University students with higher perceived physical literacy may be more likely to develop positive sport-related attitudes and sustained behavioral engagement, through which they accumulate experiences of self-management and improve attentional control and emotional regulation, thereby reducing inefficient depletion of self-control resources (Edwards et al., 2017; Li et al., 2022; Ming et al., 2018; Whitehead, 2010). At the same time, psychological resilience, defined as the capacity for positive adaptation and recovery in the face of pressure, challenge, and setback, has also been identified as an important protective factor against procrastination (Huang et al., 2023; Zhang et al., 2024). Lower self-control resource depletion may help individuals maintain more stable emotional functioning and stronger stress-coping capacity, thereby enhancing resilience (Muraven et al., 1998; Tangney et al., 2004; Tice et al., 2007); in turn, greater resilience may enable university students to remain persistent and action-oriented when confronted with difficult academic tasks and evaluative pressures, reducing procrastination arising from fear, anxiety, and ineffective coping (Huang et al., 2023; Sirois and Pychyl, 2013; Steel, 2007). Taken together, these findings suggest that perceived physical literacy may affect academic procrastination indirectly by reducing self-control resource depletion, enhancing psychological resilience, and further exerting a sequential influence through both mechanisms.

Based on the above considerations, the present study focused on university students in Shanghai and examined the effect of perceived physical literacy on academic procrastination, with particular attention to the sequential mediating roles of self-control resource depletion and psychological resilience. By approaching academic procrastination from the perspective of sport-related psychological and behavioral development, this study seeks to clarify its underlying formation pathway and to provide both theoretical support and practical implications for university-based interventions aimed at improving academic functioning and mental health through the enhancement of perceived physical literacy.

## 2 Literature review

### 2.1 Perceived physical literacy and academic procrastination

With regard to the conceptualization of perceived physical literacy, the literature has mainly approached the construct from two complementary perspectives: the promotion of health through physical activity and the holistic development of the individual. Whitehead defined physical literacy as the integration of motivation, confidence, physical competence, knowledge, and understanding that enables individuals to value and maintain appropriate levels of physical activity throughout the life course (Whitehead, 2010). This definition highlights that physical literacy concerns not only participation in movement itself, but also individuals’ understanding of the value of physical activity and their sense of responsibility for sustained engagement. Building on this view, subsequent studies have operationalized perceived physical literacy as a subjective perceptual construct reflecting individuals’ evaluations of their own movement competence, health-related knowledge, appreciation of physical activity, and capacity to interact effectively with movement environments (Sum et al., 2018; Sum et al., 2016). Scale-development research has further suggested that perceived physical literacy is multidimensional, with core dimensions including knowledge and understanding, self-expression and communication with others, and sense of self and self-confidence (Fu et al., 2025; Sum et al., 2018; Sum et al., 2016; Yang, 2016). Taken together, perceived physical literacy in the present study is defined as an individual’s systematic subjective evaluation of their sport skill mastery, understanding of health knowledge, perceived value of participation in physical activity, and adaptive capacity in movement-related contexts.

The conceptual content of perceived physical literacy suggests that the acquisition of movement skills through sport participation, together with the formation of positive cognitions about physical activity, constitutes the logical starting point for its development. Prior research has shown that participation in physical activity is a key carrier of embodied experiences through which physical literacy is cultivated, and that perceived physical literacy is positively associated with actual physical activity engagement (Ming et al., 2018). From the perspective of embodied cognition, cognition is not a passive representation of a pre-given world; rather, it is generated through action and grounded in bodily engagement with the environment (Varela et al., 1991; Wilson, 2002). From this perspective, perceived physical literacy may shape subsequent behavioral decision-making by restructuring the cognitive schemas formed in sport and movement practice.

More specifically, a higher level of perceived physical literacy may strengthen individuals’ judgments of competence and confidence in coping with tasks. When students develop a cognitive linkage between movement competence and personal efficacy, such positive appraisals may extend beyond the physical domain and support more adaptive functioning in other life contexts. In addition, the procedural experiences accumulated through sport participation, such as goal decomposition, progress monitoring, persistence, and self-regulation, may be transferable to academic contexts and help students manage complex learning tasks more effectively (Ren et al., 2021; Zhuan et al., 2024). From the perspective of emotional regulation, the development of movement-related cognition may also facilitate the formation of a positive linkage between bodily competence and psychological resilience, thereby promoting more adaptive coping strategies when students face academic demands (Ren et al., 2021). Consistent with this reasoning, research has shown that physical literacy is positively associated with resilience and mental health among college students, and that resilience partially mediates the relationship between physical literacy and mental health (Ma et al., 2021).

Further evidence suggests that physical literacy may exert broader positive psychological effects rather than merely reflecting the accumulation of motor skills or sport-related knowledge. For example, a recent study of college students with obesity found that physical literacy partially mediated the relationship between grit and well-being, with the “interaction with the environment” dimension showing the strongest mediating effect (Liu et al., 2025). This finding implies that physical literacy is also manifested in individuals’ adaptive capacity, environmental connectedness, and the tendency to sustain positive affect in physically active settings. Based on these considerations, it can be inferred that students with higher levels of perceived physical literacy are more likely to develop more positive task-related cognitions, more stable emotion-regulation capacities, and stronger self-regulatory functioning in academic contexts, thereby reducing avoidance tendencies and procrastinatory behavior. Given that academic procrastination is widely regarded as a typical form of self-regulatory failure (Steel, 2007), these mechanisms provide a plausible basis for linking perceived physical literacy to lower levels of procrastination. Accordingly, the present study proposes the following hypothesis:

*H1*: Perceived physical literacy negatively predicts academic procrastination among university students.

### 2.2 Self-control resource depletion, perceived physical literacy and academic procrastination

Previous research suggests that academic procrastination among university students is not the product of a single factor, but rather a form of self-regulatory imbalance that gradually develops under the combined influence of multiple academic goals, increasing task demands, attentional distraction, and external competitive pressure (Sirois and Pychyl, 2013; Steel, 2007). Compared with explanations that focus only on external antecedents, greater attention should be paid to the depletion of internal psychological resources caused by these persistent disturbances. According to the strength model of self-control, self-control depends on a limited pool of psychological resources, which are consumed when individuals suppress impulses, regulate emotions, maintain goal-directed behavior, and persist in task completion. When prior acts of regulation consume too many of these resources, subsequent self-control performance temporarily declines, resulting in what has been termed ego depletion (Baumeister Roy et al., 1998; Muraven et al., 1998).

In academic settings, one important reason why self-control resource depletion may induce procrastination is that procrastination is fundamentally a form of self-regulation failure. Research has shown that procrastination is not merely a matter of poor time management, but is also closely related to individuals’ tendency to prioritize short-term mood repair when confronted with aversive tasks. When learning tasks evoke anxiety, stress, or frustration, students may be more likely to alleviate immediate negative emotions than to persist in long-term goal-directed engagement. In this sense, procrastination can be understood as a self-control failure driven by the priority of short-term emotion regulation, whereby individuals delay intended tasks in order to temporarily escape discomfort (Sirois and Pychyl, 2013; Steel, 2007).

Furthermore, although self-control resources are limited, they are not necessarily irrecoverable. Some studies have shown that positive affect and other beneficial psychological resources may, to some extent, offset the adverse consequences of depletion and facilitate subsequent recovery of self-regulation (Tice et al., 2007). Consistent with this view, research on physical activity also provides empirical support for the pathway linking positive sport-related experience, enhanced self-control, and reduced procrastination. For example, researchers found among Chinese university students that physical activity significantly and positively predicted self-control and significantly and negatively predicted academic procrastination, with self-control serving as a significant mediator (Li et al., 2022). This finding suggests that sustained engagement in physical activity may not only strengthen behavioral persistence and resistance to distraction, but may also improve emotional states and perceived control, thereby increasing the self-regulatory resources available for academic tasks.

Based on the above theoretical and empirical evidence, the present study further proposes that perceived physical literacy may be an important antecedent of self-control resource depletion among university students. Perceived physical literacy reflects not only individuals’ subjective evaluations of their sport competence, health knowledge, and the value of physical activity, but also their sense of competence, task mastery, and understanding of the meaning of positive behavior in physically active contexts (Edwards et al., 2017; Fu et al., 2025; Sum et al., 2018; Sum et al., 2016). Individuals with higher perceived physical literacy are more likely to develop positive bodily self-perceptions, more stable goal-persistence awareness, and more adaptive coping styles through sport participation, which may help them preserve more sufficient self-regulatory resources when facing academic pressure. By contrast, those with lower perceived physical literacy may lack the competence-related experiences and adaptive behavioral schemas supported by embodied practice, making them more vulnerable to regulatory fatigue and resource exhaustion when dealing with academic stress and negative emotions, and consequently more likely to cope through avoidance and delay (Pignatiello et al., 2018; Tice et al., 2007; Vohs and Heatherton, 2010). Although direct empirical evidence on the relationship between perceived physical literacy and self-control resource depletion remains limited, the strength model of self-control, the mood-regulation account of procrastination, and prior findings on physical activity, self-control, and academic procrastination together provide a reasonable basis for inferring that self-control resource depletion may mediate the relationship between perceived physical literacy and academic procrastination among university students (Baumeister Roy et al., 1998; Li et al., 2022; Sirois and Pychyl, 2013). Accordingly, the present study proposes the following hypothesis:

*H2*: Self-control resource depletion mediates the relationship between perceived physical literacy and academic procrastination among university students.

### 2.3 Psychological resilience, perceived physical literacy and academic procrastination

Academic procrastination among university students cannot be attributed merely to poor time management; rather, it is also closely related to individuals’ psychological adaptability in complex academic contexts (Sirois and Pychyl, 2013; Steel, 2007). After entering university, students are often required to shift from a single examination-oriented goal to multiple developmental goals, while simultaneously coping with intertwined pressures related to academic tasks, self-planning, interpersonal adjustment, and future career development. Under such circumstances, students may be more vulnerable to psychological fluctuations when facing goal conflicts and task choices (Credé and Niehorster, 2012; Dyson and Renk, 2006). When individuals lack sufficient psychological adjustment capacity, they may be more likely to cope with academic pressure through avoidance and delay (Sirois and Pychyl, 2013). In this sense, psychological resilience, as an important psychological resource that enables individuals to maintain stable functioning and achieve positive adaptation under stress, setbacks, and uncertainty, is highly relevant to understanding the formation of academic procrastination. Psychological resilience is widely conceptualized as a dynamic process of positive adaptation in the context of adversity, rather than as a fixed personality trait (Luthar et al., 2010; Masten, 2001). The dynamic perspective on resilience further suggests that resilience develops through ongoing interactions between the individual and the environment. Existing research indicates that physical activity is an important pathway for fostering resilience among university students and young people, and recent work has shown that physical literacy is positively associated with resilience and mental health in college and adolescent samples (Ma et al., 2021; Zhu et al., 2025). More specifically, a higher level of perceived physical literacy implies that individuals are more likely to develop a positive bodily identity, stable experiences of competence, and more constructive interpretations of challenging situations through sport participation. Such positive perceptions may not only enhance confidence in dealing with difficulties, but also help individuals accumulate psychological experiences of persistence, regulation, recovery, and adaptation through repeated movement practice, thereby strengthening resilience. In other words, perceived physical literacy contributes not only to positive evaluations of physical activity itself, but also to a relatively stable expectation that “I am able to cope with pressure, regulate my state, and restore balance” (Leung et al., 2025; Ma et al., 2021).

At the same time, psychological resilience appears to play an inhibitory role in academic procrastination among university students. Individuals with higher resilience are generally better able to maintain emotional stability and goal persistence when confronted with academic pressure, task difficulty, and negative emotions, and are therefore more likely to adopt active coping and problem-solving strategies rather than avoidance and delay (Guo et al., 2024; Huang et al., 2023; Huang et al., 2022). By contrast, students with lower resilience may be more prone to cognitive withdrawal, emotional dysregulation, and behavioral delay when facing academic setbacks or demanding tasks, thereby increasing their risk of academic procrastination. These findings suggest that psychological resilience is not only an important psychological resource for adapting to academic challenges, but also a plausible mediating mechanism linking positive sport-related cognitions with academic behavioral outcomes.

Based on the above reasoning, it can be inferred that university students with higher perceived physical literacy are more likely to enhance their psychological resilience through positive movement experiences and more stable psychophysiological adaptation processes. In turn, higher psychological resilience may help them maintain emotional stability, strengthen stress coping, and sustain engagement in academic contexts, thereby reducing academic procrastination. Accordingly, the present study proposes the following hypothesis:

*H3*: Psychological resilience mediates the relationship between perceived physical literacy and academic procrastination among university students.

### 2.4 The chain mediating role of self-control resource depletion and psychological resilience between perceived physical literacy and academic procrastination

Existing research suggests that self-control resource status is closely linked to psychological resilience. When individuals are able to maintain relatively sufficient self-regulatory resources, they are more likely to preserve emotional stability, goal persistence, and positive adaptation under stressful conditions; by contrast, chronic depletion of self-control resources may undermine their capacity to cope with challenges and restore psychological balance. According to the strength model of self-control, self-control is regarded as a limited psychological resource. Once this resource reservoir is diminished, individuals’ abilities in behavioral monitoring, emotion management, and sustained task engagement are likely to be impaired, thereby increasing the risk of self-regulation failure (Baumeister Roy et al., 1998; Muraven et al., 1998). For university students, this mechanism is particularly relevant, because the college years represent both a critical developmental period for self-regulatory capacity and a stage in which self-control resources are frequently mobilized to cope with multiple academic goals and complex environmental demands.

On this basis, perceived physical literacy may be viewed as an important antecedent of self-control resource status. University students with higher perceived physical literacy are generally more likely to develop positive behavioral patterns such as regular exercise, proactive health management, and sustained self-regulation, all of which may help reduce disorderly daily resource expenditure and support a relatively stable reserve of self-control resources. Previous research has shown that regular physical activity can enhance self-control and reduce academic procrastination, with self-control playing a significant mediating role in this association (Li et al., 2022). At the same time, perceived physical literacy has been found to be positively associated with resilience and mental health in college students (Ma et al., 2021). Taken together, these findings suggest that a higher level of perceived physical literacy may reflect not only positive evaluations of movement competence and health knowledge, but also a lifestyle foundation that is more conducive to resource maintenance and behavioral organization. Conversely, when perceived physical literacy is relatively low, individuals may find it more difficult to build stable self-regulatory patterns through positive embodied experiences, which may render them more vulnerable to persistent resource depletion.

Furthermore, self-control resources are relevant not only to task execution itself, but also to whether individuals are able to effectively activate the adaptive mechanisms underlying psychological resilience. Psychological resilience is not a static trait operating independently of specific psychological resources; rather, it is a dynamic process of adaptation manifested when individuals mobilize abilities such as cognitive reappraisal, emotion regulation, and goal persistence under stress, frustration, and uncertainty (Luthar et al., 2010; Masten, 2001). In this sense, lower levels of self-control resource depletion may preserve the psychological energy necessary for the maintenance and functioning of resilience, enabling students to adopt more constructive coping strategies when confronted with academic difficulties and negative emotions rather than falling into avoidance, withdrawal, or defensive delay. Correspondingly, if individuals remain in a chronically depleted state, they may be more likely to develop negative self-evaluations and threat-oriented task perceptions—for example, underestimating their ability to complete tasks or exaggerating the pressure associated with academic demands—which may in turn weaken recovery capacity and sustained engagement, ultimately inhibiting the positive functioning of resilience. Consistent with this reasoning, recent evidence has shown a robust positive association between self-control and psychological resilience among college students (Wang et al., 2025; Zhao et al., 2025).

At the level of the outcome variable, psychological resilience has been regarded as an important protective factor against academic procrastination. University students with higher resilience are generally better able to maintain goal commitment, behavioral persistence, and adaptive adjustment in the face of task pressure and negative emotions, and are therefore less likely to rely on procrastination to relieve short-term discomfort. In contrast, when resilience is insufficient, individuals may be more likely to delay task initiation, avoid execution, or adopt passive coping strategies when confronted with challenging academic tasks (Guo et al., 2024; Huang et al., 2023). Accordingly, in the relationship between perceived physical literacy and academic procrastination, self-control resource depletion and psychological resilience may not operate as two isolated mediators; rather, they may constitute a sequential pathway with an internally progressive structure. Specifically, a higher level of perceived physical literacy may first help reduce self-control resource depletion, which in turn provides a resource foundation for the maintenance and enhancement of psychological resilience, and ultimately lowers the likelihood of academic procrastination among university students. Accordingly, the present study proposes the following hypothesis:

*H4*: Self-control resource depletion and psychological resilience sequentially mediate the relationship between perceived physical literacy and academic procrastination among university students.

## 3 Methods

### 3.1 Participants and procedures

Following approval from the Ethics Committee of Shanghai University (No. ECSHU 2025–019), this study used a convenience sampling method to recruit undergraduate students from two comprehensive universities in Shanghai, China. Both universities offered public physical education courses across different undergraduate year levels. Shanghai was chosen as the study setting because of its concentration of higher education institutions and relatively diverse student population, which helped broaden sample coverage. Moreover, students at comprehensive universities share comparable academic and daily-life contexts, providing an appropriate setting for examining the relationships among the variables of interest. Data were collected through the Wenjuanxing online survey platform. Questionnaires were distributed using two approaches. The first was centralized administration in public physical education classes. The research team communicated in advance with the physical education teaching groups at the two universities and provided unified training to the course instructors regarding the study purpose, questionnaire completion procedures, and administration precautions. When forwarding the survey link, instructors emphasized that participation was entirely voluntary. The second approach was open recruitment through active campus-based platforms, including sports clubs, interest groups, and university forums, in order to reach a broader range of student communities. Before completing the questionnaire, all participants were required to read and sign an electronic informed consent form online, indicating that they had been informed of the study purpose, the anonymous nature of the survey, and the principle of voluntary participation, and that they could withdraw from the survey at any stage without penalty. The average completion time for each questionnaire was approximately 8 min.

A total of 1,080 complete questionnaires were obtained. After excluding responses with outliers or inconsistent answers on repeated items, 951 valid questionnaires were retained for the final analysis, yielding an effective response rate of 88.06%. Based on the survey platform records and the timing information from the centralized administration sessions, the vast majority of valid responses were judged to have been collected during public physical education classes. In the final sample, 470 participants were male (49.42%) and 481 were female (50.58%). The distribution by academic year was as follows: 261 first-year students (27.44%), 283 s-year students (29.76%), 191 third-year students (20.09%), and 216 fourth-year students (22.71%). In terms of disciplinary categories, 410 participants (43.11%) were from the humanities, social sciences, philosophy, and economics, 432 (45.43%) were from science and engineering, and 109 (11.46%) were from other disciplines.

### 3.2 Measurement tools

#### 3.2.1 Perceived physical literacy scale

The Perceived Physical Literacy Instrument was initially developed by scholars from Hong Kong, China in 2016 as an assessment tool based on self-reporting (Sum et al., 2018). This scale aims to measure the self-perception of physical literacy in terms of self-awareness and confidence, self-expression and communication with others, knowledge and understanding (Sum et al., 2018). The scale consisted of 15 items in total, with sample items such as “I possess self-management skills for maintaining health” and “I actively uphold fairness and justice in sports participation.” A five-point Likert scale was employed, with scores ranging from 1 to 5, corresponding to “completely non-compliant” to “completely compliant.” The results of the confirmatory factor analysis indicated a good model fit: RMSEA = 0.024, IFI = 0.992, CFI = 0.992, and CMIN/*df* = 1.551. In this study, the Cronbach’s *α* for the scale was 0.946.

#### 3.2.2 College students’ academic procrastination scale

The Academic Procrastination Scale developed by Aitken (1982) was utilized in this study (Aitken, 1982), which has been validated by Chinese scholars for measuring the academic procrastination behaviors of college students (Chen et al., 2008). The scale consisted of 19 items in total. Sample items included “Over the past month, I usually attended classes on time” and “Over the past month, I tended to delay for a long time before starting a task.” Among these items, 9 (specifically, items 2, 4, 7, 11, 12, 14, 16, 17, and 18) are reverse-scored. Utilizing a 5-point Likert scoring method, a higher score on the questionnaire indicates a greater severity of academic procrastination. In this study, the Cronbach’s *α* for this scale was 0.888, with RMSEA = 0.023, IFI = 0.990, CFI = 0.990, and CMIN/*df* = 1.523, demonstrating robust reliability and validity.

#### 3.2.3 Self-control resource depletion scale

The simplified Self-Depletion Scale developed by Lanai, Johnson and Barnes (Lanaj et al., 2014) was utilized in this study. The scale consisted of 5 items in total. Sample items included “Over the past month, I felt exhausted” and “Over the past month, I had to exert considerable effort to concentrate on a task.” This scale employs a 5-point Likert scoring method, where a higher total score indicates a greater severity of self-loss. Previous research has demonstrated that this scale possesses strong reliability and validity, making it suitable for use with Chinese participants (Zhang et al., 2017). In this study, the Cronbach’s α for this scale was found to be 0.928, with RMSEA = 0.028, IFI = 0.992, CFI = 0.990, and CMIN/*df* = 1.523, indicating good reliability and validity.

#### 3.2.4 Psychological resilience scale

We used the Chinese revised version of Connor-Davidson Resilience Scale (Yu and Zhang, 2007), which was developed by Connor and Davidson (2003), which comprises 25 items, such as “Over the past month, I was able to think clearly under pressure” and “Over the past month, I was not easily discouraged by failure.” This scale employs a 5-point Likert scoring method, where a higher score indicates greater resilience. Since its introduction, this scale has demonstrated strong applicability in China (Chen et al., 2021). In this study, the Cronbach’s α for the scale is 0.968, RMSEA = 0.024, IFI = 0.987, CFI = 0.987, and CMIN/*df* = 1.563, indicating good reliability and validity.

### 3.3 Data processing and common method bias test

Data analysis was conducted using SPSS 29.0 statistical software and AMOS 26.0. SPSS 29.0 was employed to perform descriptive statistics and Pearson’s bivariate correlation analyses to examine the relationships among the variables. AMOS 26.0 was used to construct the structural equation model and to further test the path relationships and sequential mediating effects. The model fit indices indicated a good fit to the data, with goodness-of-Fit Index (GFI) = 0.965, root mean squared error of approximation (RMSEA) = 0.016, normed Fit Index (NFI) = 0.964, and CMIN/*df* = 1.246, all of which met the recommended criteria for acceptable model fit. The mediating effects were tested using the bias-corrected Bootstrap method with 5,000 resamples to generate 95% confidence intervals (CIs). A mediating effect was considered significant when the confidence interval did not include zero.

Common method bias was assessed using Harman’s single-factor test. The results of the unrotated principal component analysis showed that nine factors with eigenvalues greater than 1 were extracted, and the first factor accounted for 20.988% of the total variance, which was below the 40% threshold, indicating that common method bias was not a serious concern in this study. In addition, the variance inflation factor (VIF) values for the core variables ranged from 1.061 to 1.137, all of which were below 5, suggesting that no serious multicollinearity problem was present.

## 4 Results

### 4.1 Descriptive statistics and correlation analysis

Correlation analysis (Table 1) showed that perceived physical literacy was significantly negatively correlated with academic procrastination and self-control resource depletion, and significantly positively correlated with psychological resilience, with correlation coefficients of −0.176, −0.121, and 0.188, respectively (all *p* < 0.01). Academic procrastination was significantly positively correlated with self-control resource depletion (*r* = 0.287, *p* < 0.01) and significantly negatively correlated with psychological resilience (*r* = −0.208, *p* < 0.01). In addition, self-control resource depletion was significantly negatively correlated with psychological resilience (*r* = −0.196, *p* < 0.01). The square root of the AVE for each construct was greater than the absolute value of its correlations with other constructs, and the AVE for each construct exceeded 0.50, indicating that the measurement model demonstrates good discriminant and convergent validity.

**Table 1:** Correlation analysis and the square root of AVE.

Variable	M	SD	1	2	3	4
1. Perceived physical literacy	3.293	0.692	**0.597** ^#^			
2. Academic procrastination	3.036	0.816	−0.176**	**0.731** ^#^		
3. Self-control resource depletion	2.670	0.825	−0.121**	0.287**	**0.719** ^#^	
4. Psychological resilience	3.319	0.657	0.188**	−0.208**	−0.196**	**0.610** ^#^

**p < 0.01. ^#^Bolded values indicate the square roots of the average variance extracted (AVE).

### 4.2 Pathway analysis

The structural equation model was used to examine the mechanism through which perceived physical literacy influences academic procrastination among university students. The results (Table 2) showed that the direct effect of perceived physical literacy on academic procrastination was significant, and that self-control resource depletion and psychological resilience constituted significant mediating pathways in this relationship. Specifically, perceived physical literacy significantly and negatively predicted self-control resource depletion (*β* = −0.160, SE = 0.053, CR = − 3.881, *p* < 0.001), indicating that higher levels of perceived physical literacy were associated with lower levels of self-control resource depletion. Perceived physical literacy also significantly and positively predicted psychological resilience (*β* = 0.225, SE = 0.042, CR = 5.021, *p* < 0.001), suggesting that perceived physical literacy had a positive effect on individuals’ psychological resilience. Meanwhile, self-control resource depletion significantly and negatively predicted psychological resilience (*β* = −0.217, SE = 0.030, CR = − 5.155, *p* < 0.001), indicating that greater self-control resource depletion was associated with lower psychological resilience.

**Table 2:** Path coefficients of perceived physical literacy affecting academic procrastination.

Path	Standard path coefficient	Non-standard path coefficient	Standard error (SE)	Critical ratio (CR)	*p*
Perceived physical literacy → self-control resource depletion	−0.16	−0.207	0.053	−3.881	*p*<0.001
Perceived physical literacy → psychological resilience	0.225	0.210	0.042	5.021	*p*<0.001
Self-control resource depletion → psychological resilience	−0.217	−0.157	0.030	−5.155	*p*<0.001
Self-control resource depletion → academic procrastination	0.265	0.285	0.041	7.012	*p*<0.001
Perceived physical literacy → academic procrastination	−0.127	−0.176	0.054	−3.28	*p* = 0.001
Psychological resilience → academic procrastination	−0.143	−0.213	0.06	−3.538	*p*<0.001

At the outcome level, self-control resource depletion significantly and positively predicted academic procrastination among university students (*β* = 0.265, SE = 0.041, CR = 7.012, *p* < 0.001), indicating that higher levels of self-control resource depletion were associated with greater academic procrastination. Psychological resilience significantly and negatively predicted academic procrastination (*β* = −0.143, SE = 0.060, CR = − 3.538, *p* < 0.001). In addition, perceived physical literacy continued to significantly and negatively predict academic procrastination (*β* = −0.127, SE = 0.054, CR = − 3.280, *p* = 0.001), suggesting that even after self-control resource depletion and psychological resilience were included in the model, perceived physical literacy retained a substantial direct effect on academic procrastination.

**Figure 1:**
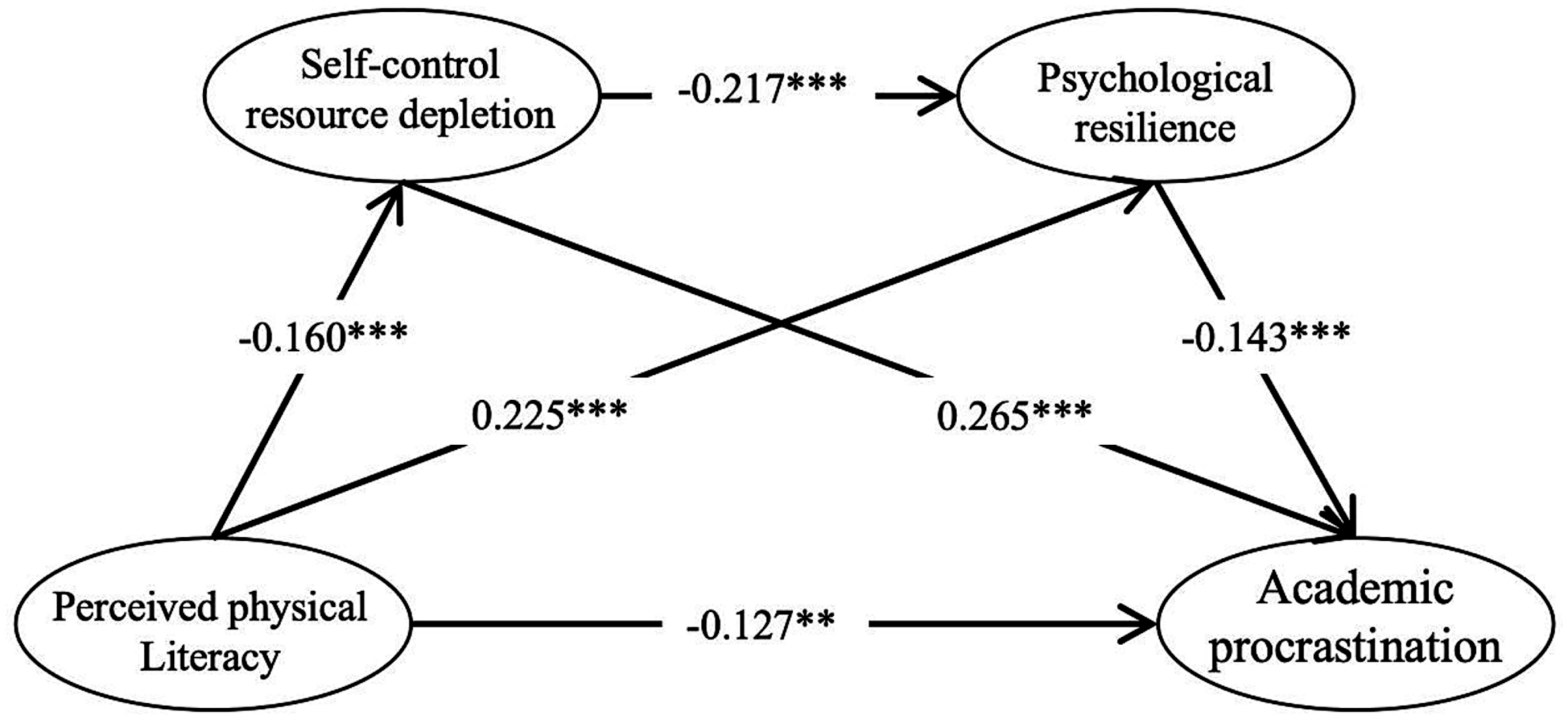
Role of self-control resource depletion and psychological resilience in the relationship between perceived physical literacy and academic procrastination. ^**^p < 0.01, ^***^p < 0.001.

Overall, perceived physical literacy not only directly and negatively predicted academic procrastination among university students, but also indirectly influenced academic procrastination by reducing self-control resource depletion, enhancing psychological resilience, and exerting a sequential effect through self-control resource depletion and psychological resilience (Figure 1).

### 4.3 Mediating effect analysis

The results (Table 3) showed that, in the relationship between perceived physical literacy and academic procrastination among university students, both self-control resource depletion and psychological resilience played significant mediating roles, and together formed a significant sequential mediation pathway. Specifically, the indirect effect of perceived physical literacy on academic procrastination through self-control resource depletion was −0.042, with a 95% confidence interval (CI) of [−0.069, −0.020], accounting for 20.39% of the total effect. The indirect effect through psychological resilience was −0.032, with a 95% CI of [−0.058, −0.015], accounting for 15.53% of the total effect. In addition, the sequential indirect effect through the pathway of self-control resource depletion → psychological resilience was −0.005, with a 95% CI of [−0.012, −0.002], accounting for 2.43% of the total effect. Because the confidence intervals for all of the above indirect pathways did not include zero, all mediating effects were statistically significant. The total effect was −0.206, with a 95% CI of [−0.277, −0.132]. These findings indicate that perceived physical literacy not only directly and negatively predicted academic procrastination among university students, but also indirectly reduced academic procrastination by lowering self-control resource depletion, enhancing psychological resilience, and exerting a sequential effect through the pathway from self-control resource depletion to psychological resilience.

**Table 3:** Mediating effects analysis.

Mediating pathway	Effect	Effect size	Lower limit of the confidence interval (LLCI)	Upper limit of the confidence interval (ULCI)	Effect proportion
Perceived physical literacy → self-control resource depletion → academic procrastination	Mediating effect	−0.042	−0.069	−0.02	20.39%
Perceived physical literacy → psychological resilience → academic procrastination	Mediating effect	−0.032	−0.058	−0.015	15.53%
Perceived physical literacy → self-control resource depletion → psychological resilience → academic procrastination	Mediating effect	−0.005	−0.012	−0.002	2.43%
Perceived physical literacy → self-control resource depletion → psychological resilience → academic procrastination	Total effect	−0.206	−0.277	−0.132	100%

## 5 Discussion

This study found that higher levels of perceived physical literacy were associated with lower self-control resource depletion, higher psychological resilience, and, ultimately, lower levels of academic procrastination among university students. These findings suggest that perceived physical literacy is not only an important reflection of students’ sport-related cognition and value judgments, but also a meaningful psychological resource that may shape their learning behavior and adaptive functioning. This interpretation is broadly consistent with previous evidence showing that physical literacy is positively associated with resilience and mental health in college students, and that the broader literature on perceived physical literacy in tertiary education has increasingly linked it to positive mental health outcomes (Ma et al., 2021).

Previous studies have primarily focused on the beneficial effects of sport participation and physical activity on positive psychological qualities among university students. By contrast, research on academic procrastination has more often examined the problem from the perspectives of self-regulatory failure, short-term mood repair, and, more recently, digital addiction-related factors such as smartphone overuse. Although these lines of inquiry have generated important findings, relatively little attention has been paid to perceived physical literacy as a more holistic and system-level cognitive-evaluative construct. Without such a perspective, it is difficult to reveal the deeper cognitive foundations underlying the development of positive psychological qualities, and it is also harder to refine the theoretical explanation of how academic procrastination develops among university students (Chen and Lyu, 2024; Ma et al., 2021; Sirois and Pychyl, 2013; Steel, 2007). In particular, empirical evidence remains limited regarding how perceived physical literacy exerts its influence on academic procrastination through specific psychological mechanisms.

Against this backdrop, the present study integrated the limited-resource model of self-control with the dynamic model of resilience and, from the perspective of perceived physical literacy, further examined both the logic underlying self-control resource depletion and the process through which individuals adapt psychologically to environmental stress. The results indicate that perceived physical literacy may influence academic procrastination through the sequential mediating roles of self-control resource depletion and psychological resilience. This finding not only extends the theoretical boundary of research on the relationship between perceived physical literacy and academic procrastination, but also offers a practical pathway for improving students’ academic self-management and informing university-based mental health interventions and sport-for-development practices. Existing evidence that physical activity is linked to lower academic procrastination through self-control, together with evidence connecting physical literacy, resilience, and mental health, provides support for this interpretation (Li et al., 2022; Ma et al., 2021).

### 5.1 The impact of perceived physical literacy on academic procrastination

The findings of this study indicate that perceived physical literacy negatively predicts academic procrastination among university students, thereby supporting Hypothesis 1. This suggests that higher levels of perceived physical literacy are associated with lower levels of academic procrastination. In general, this result is consistent with previous research. Earlier studies have mainly taken sport participation as the point of entry and examined its influence on procrastinatory behaviors in students’ academic and daily lives, with most concluding that participation in physical activity can, to some extent, reduce procrastination among university students (Li et al., 2022).

However, unlike previous research, which has primarily focused on the behavioral dimension of whether students participate in physical activity, the present study conceptualized perceived physical literacy as a more holistic and systematic cognitive-psychological construct. Specifically, perceived physical literacy reflects not only students’ judgments of their own sport skill competence, but also their subjective evaluations of health knowledge, the value of sport participation, and their capacity to adapt to physically active contexts (Sum et al., 2018; Sum et al., 2016; Whitehead, 2010). The reason why perceived physical literacy can predict academic procrastination lies precisely in the fact that it is not merely a simple evaluation of whether one “can exercise,” but rather a comprehensive cognitive representation grounded in long-term embodied experiences of physical activity. Therefore, although the present study is consistent with previous research in showing that sport-related factors can help inhibit academic procrastination, the two differ substantially in terms of variable content and underlying mechanisms. More specifically, sport participation primarily reflects overt behavioral engagement, whereas perceived physical literacy further captures individuals’ overall cognition and value judgments regarding the meaning of sport, their own competence, and healthy lifestyles (Sum et al., 2016; Whitehead, 2010).

For this reason, the present study not only confirms the negative predictive effect of perceived physical literacy on academic procrastination, but also provides a new perspective for further exploring the internal mechanisms through which physical literacy, as an overall cognitive evaluation, continuously influences academic behavior among university students. In other words, compared with approaches that focus solely on the frequency or intensity of physical activity, the present study contributes to a deeper understanding of how physical literacy shapes the development and alleviation of academic procrastination from the perspective of cognitive appraisal and the generation of psychological resources. At the same time, embodied cognition theory emphasizes that experiences of bodily activity can shape how individuals perceive and respond to action, effort, and environmental demands (Wilson, 2002). University students with higher levels of perceived physical literacy are more likely to have accumulated direct experiences of self-monitoring and coping with setbacks in physically active contexts. Such experiences not only strengthen their embodied belief that “I am capable of completing tasks,” but also facilitate a stronger state of action readiness and willingness to execute when facing academic tasks, thereby reducing procrastination triggered by avoidance of difficulty, fear of failure, or insufficient self-regulation. In contrast, when individuals lack a high level of perceived physical literacy, it may be more difficult for them to form a positive linkage between bodily experience and cognitive evaluation, which may in turn hinder the development of a stable behavioral initiation mechanism in academic tasks (Ma et al., 2021; Wilson, 2002).

### 5.2 The role of self-control resource depletion in the impact of perceived physical literacy on academic procrastination

This study found that self-control resource depletion mediated the relationship between perceived physical literacy and academic procrastination, indicating that perceived physical literacy may alleviate academic procrastination among university students by influencing their level of self-control resource depletion. Thus, Hypothesis 2 was supported. This finding is broadly consistent with previous studies showing that sport participation and physical activity can promote positive psychological resources among university students. In other words, both actual engagement in physical activity and individuals’ systematic perceptions of physical literacy may play an important role in shaping the process through which psychological resources are consumed. From this perspective, students’ recognition of the value of sport participation and their positive perceptions of physical literacy may contribute not only to psychological growth but also to more adaptive patterns of behavioral regulation. The added value of the present study lies in its focus on perceived physical literacy, which extends understanding of the overall functions of sport beyond behavioral participation and helps explain how the broader effects of sport may influence academic behavior through changes in individuals’ self-control resource status. In this sense, the findings further highlight the positive role of sport as both an educational resource and a value-laden developmental context in university students’ growth and development (Li et al., 2022; Ma et al., 2021).

With regard to the mechanism through which self-control resource depletion affects academic procrastination, prior research suggests that this process can be understood from the perspective of self-regulatory failure and short-term mood repair (Sirois and Pychyl, 2013; Steel, 2007). Academic procrastination often occurs when individuals are confronted with complex or highly demanding tasks, which typically require substantial cognitive and self-regulatory resources. When self-control resources are depleted through repeated use, individuals’ executive functioning, behavioral monitoring, and persistence in task engagement may be weakened, thereby increasing the likelihood of procrastination. In addition, in the context of rapid technological change and mounting competitive pressure, academic procrastination among university students has become increasingly complex: it is related not only to the intrinsic difficulty of tasks, but also to attentional fragmentation and the growing availability of immediate temptations. This pattern further supports the applicability of the limited-resource perspective on self-control in explaining academic procrastination. According to this framework, the self-control resources available to individuals at a given time are finite, and these resources are more easily exhausted under conditions of multiple competing goals and persistent distraction (Baumeister Roy et al., 1998; Sirois and Pychyl, 2013; Steel, 2007).

Based on this logic, perceived physical literacy may influence academic procrastination by shaping the way individuals allocate and preserve their self-regulatory resources. Students who maintain regular exercise habits are more likely to develop stable goal awareness and behavioral persistence through both the practice of physical activity and their recognition of its value, and perceived physical literacy may serve as an important psychological foundation for such goal stability. More specifically, when individuals transfer the rule awareness, volitional qualities, and task-persistence experiences internalized in sport contexts into the academic domain, their goal-directed behavior may become more automatically activated. This type of psychological schema may help shorten hesitation before task initiation and directly facilitate engagement, thereby reducing the excessive expenditure of willpower resources at the beginning of academic tasks. Moreover, individuals with higher perceived physical literacy may draw on the stable sense of self-efficacy cultivated through sport participation to reconstruct the way they cognitively appraise academic tasks, becoming more likely to adopt a competence-development frame rather than an error-avoidance frame. Under such a motivational orientation, academic challenges may be interpreted less as sources of threat and more as opportunities for competence demonstration and personal growth. As a result, cognitive resources may be redirected from defensive consumption toward active investment, which may buffer the resource exhaustion triggered by complex tasks. Therefore, fostering perceived physical literacy among university students may help reduce self-control resource depletion and, in turn, lower the risk of academic procrastination (Li et al., 2022; Ren et al., 2021).

### 5.3 The role of psychological resilience in the impact of perceived physical literacy on academic procrastination

This study found that psychological resilience plays a mediating role in the relationship between perceived physical literacy and academic procrastination. This indicates that perceived physical literacy can influence college students’ psychological resilience, which in turn affects academic procrastination, supporting Hypothesis 3. This finding is largely consistent with previous research. According to the dynamic model of psychological resilience, it is understood that psychological resilience is not a fixed trait; rather, it is a dynamic adaptive capacity that individuals develop through the accumulation of resources and the optimization of strategies while facing challenges (Stewart and Yuen, 2011).

Physical literacy, defined as a multi-dimensional system that includes cognitive aspects of sports, emotional experiences, and behavioral practices, serves as a buffering mechanism against stress through the continuous accumulation of psychological capital. When individuals engage in goal-oriented training and navigate setbacks in sports activities, they cultivate a reservoir of adaptive strategies. These strategies can effectively mitigate the inclination towards task avoidance when applied to the academic domain (Hobfoll, 2001). Research shows that the experience formed in sports activities can enhance the plasticity of psychological resilience, making individuals more inclined to adopt problem-focused strategies rather than emotional avoidance when facing academic pressure (Sarkar and Fletcher, 2014).

From the perspective of the cognitive-emotional regulation system, cultivating self-efficacy in sports contexts can enhance individuals’ sense of control in academic domains and reduce the perception of task-related threats (Bandura et al., 1997). Conversely, the emotional regulation benefits of sports can help break the vicious cycle of anxiety and avoidance that often accompanies academic procrastination (Sirois and Pychyl, 2013), allowing individuals to more readily activate the executive control network when confronted with academic tasks (Basso and Suzuki, 2017; Sirois et al., 2017). Moreover, because participation in physical activity often emphasizes moral norms such as self-discipline and perseverance, these norms can gradually be internalized as individuals’ behavioral principles through long-term development (Sirois and Pychyl, 2013; Sirois et al., 2017). When academic tasks align with these values, individuals are more inclined to harness intrinsic motivation rather than relying solely on limited self-control resources, thereby fostering sustainable motivation to combat procrastination (Ryan and Deci, 2000). Consequently, psychological resilience plays a crucial bridging role in the process whereby perceived physical literacy influences academic procrastination. Therefore, universities should integrate the cultivation of physical literacy with the enhancement of psychological resilience by embedding goal-setting and stress-coping modules into physical education courses and promoting an integrated in-class and extracurricular physical education system, so as to help students develop resilient mindsets in academic contexts and thereby reduce procrastination caused by insufficient psychological resilience.

### 5.4 The chain mediating role of self-control resource depletion and psychological resilience in the impact of perceived physical literacy on academic procrastination

The results of this study indicate that self-control resource depletion and psychological resilience serve as a chain mediating factor in the relationship between perceived physical literacy and academic procrastination, thereby supporting Hypothesis 4. Although prior research has not directly established a systematic pathway among perceived physical literacy, self-control resource depletion, psychological resilience, and academic procrastination, existing studies have provided evidence that self-control plays an important role in fostering psychological resilience (Huang et al., 2022; Job et al., 2010; Ozbay et al., 2007; Yang et al., 2024). Numerous studies have elucidated their internal mechanisms through the lens of the theory of limited self-control and the dynamic model of psychological resilience. The literature suggests that individuals with high perceived physical literacy can effectively self-regulate at the physiological level, allowing them to conserve cognitive resources for goal setting and execution within the academic context, rather than depleting these resources due to competition triggered by stress. Conversely, the accumulation of self-control resources lays a cognitive-emotional foundation for the enhancement of psychological resilience. According to the dual-system theory of self-control, when self-control resources are abundant, the reflective system can effectively inhibit the immediate responses of the impulsive system, thereby improving adaptive assessments of academic challenges (Strack et al., 2004). Furthermore, by reducing self-control resource depletion, enhanced psychological resilience facilitates the maintenance of goal-directed processes and the integration of social resources, thereby inhibiting academic procrastination. Research indicates that individuals with high psychological resilience actively engage their social support networks when confronted with academic challenges. This engagement alleviates the emotional burden associated with task execution by facilitating empathetic feedback and resource sharing during social interactions, thus diminishing the inclination to procrastinate (Ozbay et al., 2007; Southwick et al., 2016). Additionally, it is important to highlight that this pathway is moderated by a growth mindset (Mangels et al., 2006). The development of physical literacy fosters an individual’s belief in the malleability of their abilities. This perspective encourages individuals to perceive the depletion of self-control resources as a temporary and recoverable state, rather than a fixed limitation. Consequently, they can achieve a dynamic balance between resource renewal and behavioral adjustment through psychological resilience (Edwards et al., 2017; Ye et al., 2025).

In conclusion, there is a close relationship between perceived physical literacy, self-control resource depletion, psychological resilience and academic procrastination, and they may constitute important mechanisms influencing academic procrastination. This influence mechanism includes direct and indirect effects, that is, perceived physical literacy can not only directly affect college students’ academic procrastination, but also indirectly influence academic procrastination through the sole mediating effect of self-control resource depletion, the sole mediating effect of psychological resilience, and the chain mediating effect of self-control resource depletion and psychological resilience. Therefore, schools should encourage college students to actively participate in sports exercises, cultivate healthy behaviors, enhance sports morality, and improve physical literacy, and pay attention to adopting relevant strategies to reduce self-control resource depletion and cultivate their psychological resilience, enabling students to more effectively combat academic procrastination through sports exercises.

Therefore, universities should systematically integrate multi-dimensional physical literacy development programs into physical education and mental health curricula. Additionally, they should optimize the design of physical education courses to strengthen students’ self-regulation abilities and incorporate sports-related cognitive training into academic settings to help reduce the incidence of academic procrastination. Enhancing physical literacy through sports education not only improves students’ motor skills and health awareness but also facilitates the transfer of self-regulatory principles from sports to academics, thereby establishing goal execution inertia and an automatic task activation mode. This process reduces the depletion of self-control resources and mitigates procrastination behaviors. Furthermore, embedding goal-setting and stress management modules in sports courses can enhance psychological resilience, enabling students to adopt more adaptive and problem-focused coping strategies when facing academic stress. Collectively, these approaches underscore the potential of cultivating physical literacy as a promising pathway to improve academic self-management and reduce procrastination among university students.

## 6 Conclusion

This study investigated the effect of perceived physical literacy on academic procrastination and its underlying mechanisms among university students in Shanghai.

The findings showed that: (1) Perceived physical literacy significantly and negatively predicted academic procrastination among university students, indicating that a higher level of perceived physical literacy may effectively reduce students’ tendency to procrastinate academically. (2) Both self-control resource depletion and psychological resilience played significant mediating roles in the relationship between perceived physical literacy and academic procrastination. Specifically, perceived physical literacy influenced academic procrastination not only by reducing self-control resource depletion and enhancing psychological resilience independently, but also indirectly through the sequential pathway of self-control resource depletion → psychological resilience. (3) The effect of perceived physical literacy on academic procrastination involved both direct and multiple indirect effects, suggesting that perceived physical literacy is not only a subjective cognitive evaluation related to physical activity, but also an important psychological foundation that can influence academic behavior through the regulation of psychological resources and positive adaptation mechanisms.

Overall, this study extends the explanatory framework of academic procrastination among university students from the perspective of perceived physical literacy and reveals the internal mechanism through which perceived physical literacy affects academic procrastination via the combined roles of self-control resource depletion and psychological resilience. These findings not only enrich the theoretical understanding of how physical literacy promotes psychological development and behavioral optimization among university students, but also provide empirical support and practical implications for universities seeking to reduce academic procrastination by strengthening physical literacy cultivation, lowering psychological resource depletion, and enhancing psychological resilience.

## 7 Limitations and prospects

First, regarding the selection of the sample. This study used a convenience sampling method and recruited participants only from two comprehensive universities in Shanghai, which limits the representativeness of the sample and prevents the findings from fully reflecting the overall situation of the broader university student population. Although universities in China that offer physical education courses across all four undergraduate years are relatively uncommon, which made sample recruitment more challenging, this does not justify the limited sample scope. In addition, the sequential mediation effect identified in this study might have been stronger if a larger sample had been included. Future research could further examine the provision of physical education courses across Chinese universities and recruit participants from a broader range of institutions and student groups.

Second, regarding research methods, this study relied on self-report questionnaires and statistical analyses to examine the relationships among variables. As a result, the measures may be influenced by participants’ subjective psychological states and may not fully capture the constructs at an objective level. In addition, because physical activity behavior was not directly measured or controlled in the current dataset, future studies should include behavioral indicators of physical activity (e.g., frequency, duration, or device-based measures) as covariates to better disentangle perception/competence from behavior/frequency. Accordingly, future research is encouraged to adopt more diversified approaches, such as multi-method assessments and objective physiological/behavioral indicators, to more comprehensively quantify and clarify the relationships among these variables.

Third, due to practical constraints, this study used a cross-sectional design and therefore cannot establish causal relationships or determine temporal directionality. Future studies should employ longitudinal or intervention designs and incorporate more objective physiological and psychological measures to examine how perceived physical literacy and academic procrastination co-develop over time and to test the proposed mechanisms more rigorously.

## Data Availability

The raw data supporting the conclusions of this article will be made available by the authors, without undue reservation.
